# Myocardial performance imaging for the early identification of cardiac dysfunction in neonates with sepsis

**DOI:** 10.1007/s10554-024-03120-z

**Published:** 2024-06-22

**Authors:** Sudheshna Lalitha Sumbaraju, Krishnananda Nayak, Sridevi Prabhu, Vidya Nayak, K Prathiksha Prabhu, Leslie Edward Lewis

**Affiliations:** 1 Department of Cardiovascular Technology, Manipal College of Health Professions (MCHP), Manipal Academy of Higher Education, Manipal, India; 2 Department of Paediatrics, Kasturba Medical College, Manipal Academy of Higher Education, Manipal, India; 3 Department of Allied Health Sciences, Manipal Tata Medical College, Jamshedpur, Jharkhand, India

**Keywords:** Neonatal sepsis, Speckle tracking echocardiography, Tissue doppler imaging, Myocardial dysfunction

## Abstract

**Purpose:**

The assessment of cardiac performance in septic new-borns is crucial for detecting hemodynamic instability and predicting outcome. The aim of the study is to assess myocardial performance in neonates with sepsis for the early identification of cardiac dysfunction.

**Patients and methods:**

A case control study was carried out from September 2022 to May 2023 at the Neonatal Intensive care unit, Kasturba Medical College, Manipal. A total of 68 neonates were included in the study, with 33 females and 35 males. The study population was further subdivided into 3 groups namely preterm septic neonates (*n* = 21), term septic neonates (*n* = 10) and non-septic healthy controls (*n* = 37). The cardiac structure and function were assessed using conventional method, Tissue Doppler imaging (Sm) and speckle tracking echocardiography (GLS). The study was approved by the Institutional Ethics Committee at Kasturba Medical College, Manipal (approval number IEC: 90/2022). The CTRI registration number for the study is CTRI/2022/09/045437 and was approved on September 12, 2022. Prior to the neonate’s enrolment, informed consent was obtained from their mothers or legal guardians.

**Results:**

Out of the total 68 neonates, 31 were cases and 37 were controls which included 33 females and 35 males. LV systolic function was not statistically significant between cases and controls. E/A ratio of the mitral valve was significantly lower in septic newborns than in healthy neonates. (1.01 ± 0.35 vs 1.18 ± 0.31, p < 0.05) preterm neonates showed significantly lower Lateral E’ and RV E’ velocities than term neonates. TAPSE was significantly lower in septic preterm neonates. (8.61 ± 1.28 vs. 10.7 ± 2.11, *p* < 0.05) No significant difference was noted in the Myocardial Performance Index between septic neonates and healthy neonates. LV Global Longitudinal Strain was slightly lower in preterm septic neonates than in term neonates with sepsis.

**Conclusion:**

Septic newborns are associated with LV diastolic dysfunction, RV systolic dysfunction and substantially higher pulmonary systolic pressures.

“Neonatal sepsis is a clinical syndrome characterized by signs and symptoms of infection with or without accompanying bacteremia in the first month of life. It encompasses various systemic infections of the new born such as septicemia, meningitis, pneumonia, arthritis, osteomyelitis, and urinary tract infections.” [1].

Globally, sepsis affects 4 to 22 new-borns per 1,000 live births, with frequencies changing inversely with the gestational age at birth [2]. Furthermore, clinical sepsis is most common in India with a prevalence of 17,000 per 1,00,000 live births and has a fatality rate ranging between 25 and 65% [3].

Neonatal sepsis is classified as early and late onset sepsis on the basis of time and age of onset [4]. The effects of neonatal sepsis in neonates include cardiovascular problems, myocyte destruction, and changes in cardiac blood flow caused by inflammatory mediators [5].

Tissue Doppler imaging is more sensitive in assessing diastolic function and is less dependent on preload and afterload than conventional Doppler methods [6].

Myocardial strain and strain rate analyse cardiac deformational changes with the advantage of detecting preclinical cardiac dysfunction. Strain is a measure of myocardial deformation. Whereas Strain rate is the speed at which the deformation occurs and is expressed as per second [7].

Speckle tracking echocardiography imaging technique outlines the limitations imposed by traditional methods like M mode, volumetric methods and Doppler imaging technique which are routinely used to assess cardiac function.

Neonates with sepsis showed subclinical diastolic and systolic dysfunction when assessed using tissue Doppler imaging [8].

Strain imaging has been proved as a sensitive method in detecting sub- clinical myocardial dysfunction. However only a few studies have been done on the assessment of cardiac function in preterm and term neonates with sepsis using speckle tracking echocardiography. Thus, in this present study we aimed to assess cardiac function in septic neonates by using various non-invasive assessment methods such as Tissue Doppler Imaging, Speckle Tracking Echocardiography which would assist in detecting subclinical LV dysfunction and in determining the optimal timing for management strategies.

## Patients and methods

### Study design and patient selection

A case control study was carried out from September 2022 to May 2023 at the Neonatal Intensive Care Unit (NICU), Kasturba Medical College, Manipal. A total of 68 neonates were included in the study. The study population was further subdivided into 3 groups namely preterm septic neonates (Gestational age < 37weeks, *n* = 21), term septic neonates (Gestational age > 37weeks, *n* = 10) and non-septic healthy controls (*n* = 37). Neonates admitted to Neonatal Intensive care unit (NICU), Kasturba Medical College, Manipal with diagnosis of culture positive sepsis, Intra Uterine Growth Restriction, and Babies on ventilators and Non Invasive Ventilation were included in the study. Whereas Infants with congenital malformations, Hypoxic ischemic encephalopathy, genetic syndromes, critical CHDs were excluded. Enrolled control subjects were the ones admitted to Neonatal Intensive care unit, Manipal without culture proven sepsis.

The study was approved by the Institutional Ethics Committee, Kasturba Medical College, Manipal, (approval number IEC: 90/2022). The CTRI registration number for the study is CTRI/2022/09/045437 and was approved on September 12, 2022. Informed consent was obtained from the mothers or guardians of the neonates before their enrolment.

### Echocardiographic imaging

For the neonates fulfilling the inclusion criteria, initial Echocardiographic examination was performed within 48 h of diagnosis using Vivid IQ echo machine from GE with 5 MHz transducer. The cardiac structure and function were assessed using conventional, Tissue Doppler imaging and speckle tracking echocardiography with ECG.

**Fig. 1:**
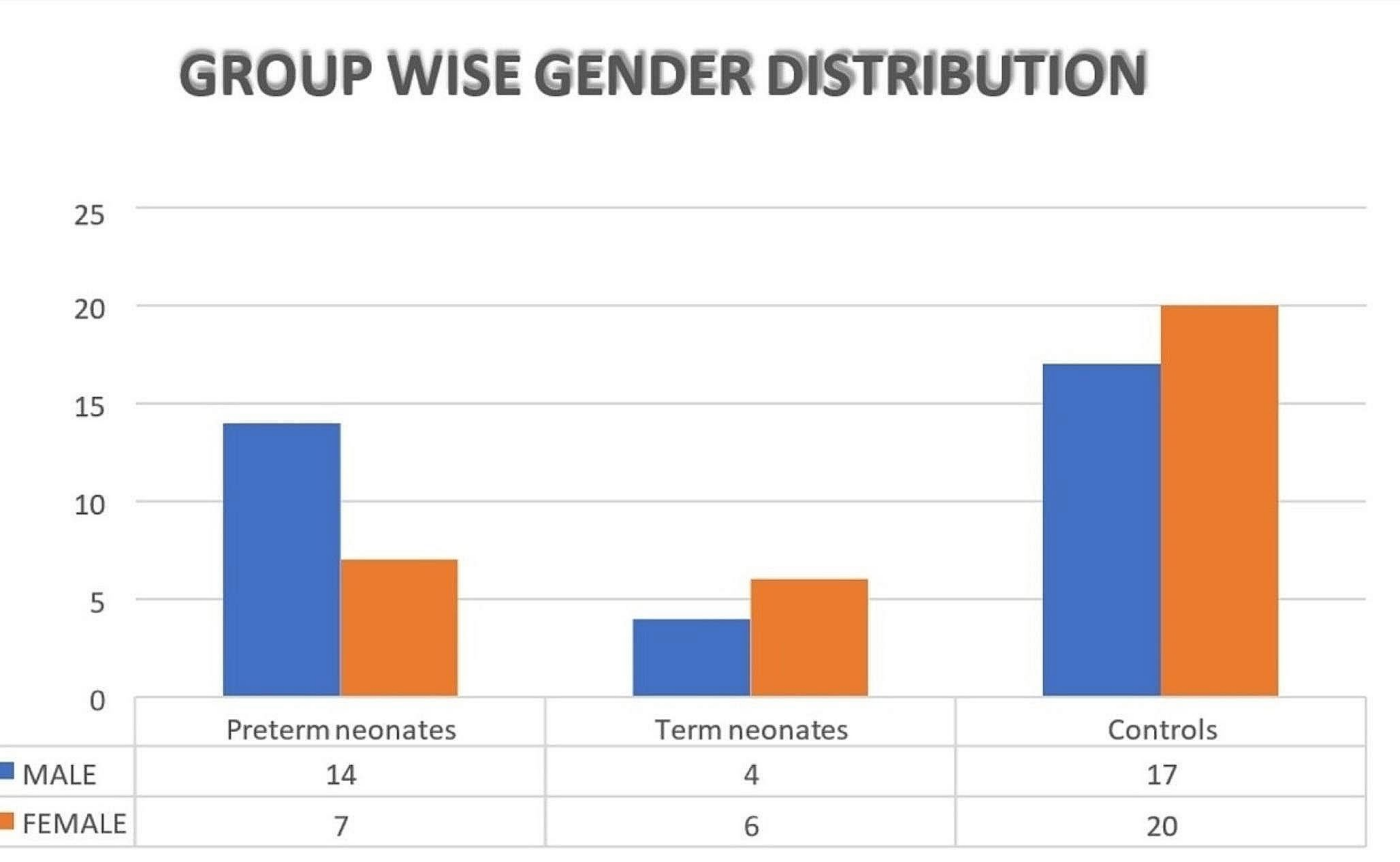
Bar graph representation of group wise gender distribution

LV internal dimensions, Interventricular septum (IVS), Posterior wall (PW) thickness, Ejection Fraction (EF), and Fractional Shortening (FS) were obtained from M mode by placing the cursor perpendicular to mitral leaflets. Mitral (E, E/A ratio) and aortic flow velocities was acquired from apical views (4 &5 chamber) respectively using pulse wave doppler by placing sample volume at tips (Fig. 1).

Tricuspid annular plane systolic excursion (TAPSE) and Inferior vena cava (IVC) values were obtained from M mode at tricuspid valve annulus and IVC respectively [7].

### Conventional doppler

The TEI-index for the left and right ventricles were determined by analysing the Doppler tracings of the respective AV valves and semilunar valves and was measured using the formula (IVRT + IVCT)/ET [7]. [IVRT: Isovolumetric relaxation time, IVCT: Isovolumetric contraction time, ET: Ejection time].

RV systolic pressure was obtained through continuous wave Doppler analysis with the sample volume across the TR jet. The obtained TR jet velocity provides an estimation of the pressure difference between the right heart chambers. To quantify pulmonary hypertension, systolic pulmonary artery pressure (PAP) was estimated by calculating the fraction of systemic systolic blood pressure (BP). A ratio of less than one-third was considered normal, while ratios ranging from one-third to two-thirds indicated mild pulmonary hypertension. Ratios between two-thirds and one signified moderate pulmonary hypertension and ratios exceeding one indicated severe pulmonary hypertension [9].

### Tissue doppler imaging

**Fig. 2:**
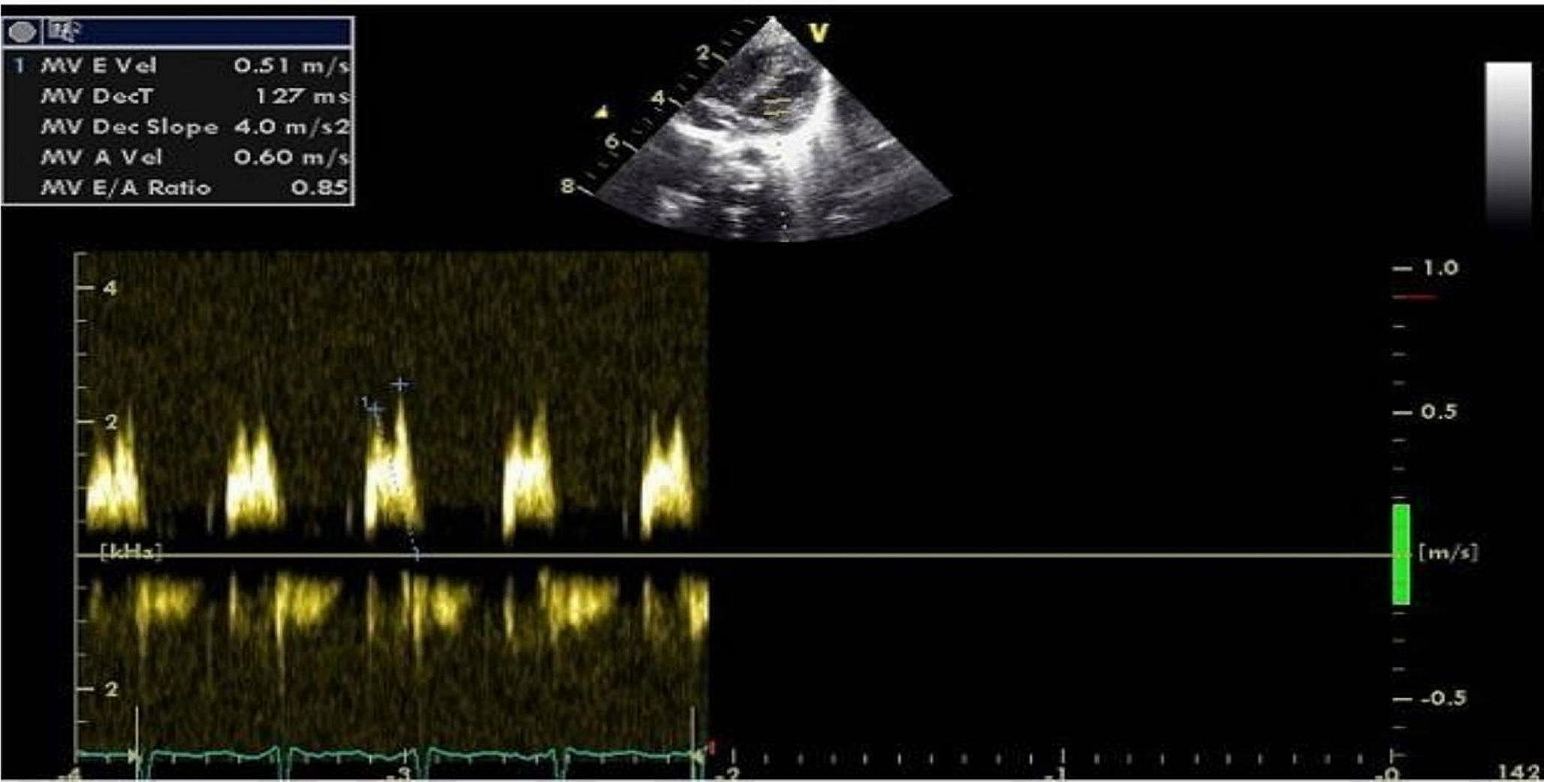
LV diastolic function by conventional doppler

TDI targets the tissue motion and has 3 phases which are denoted by early diastolic tissue annular velocity (Ea), late diastolic annular velocity (Aa), and systolic annular velocity (Sa), respectively. Tissue Doppler velocities were obtained by placing the cursor across respective walls and by applying Pulse wave Doppler [7] (Fig. 2).

### Tissue deformation imaging

**Fig. 3:**
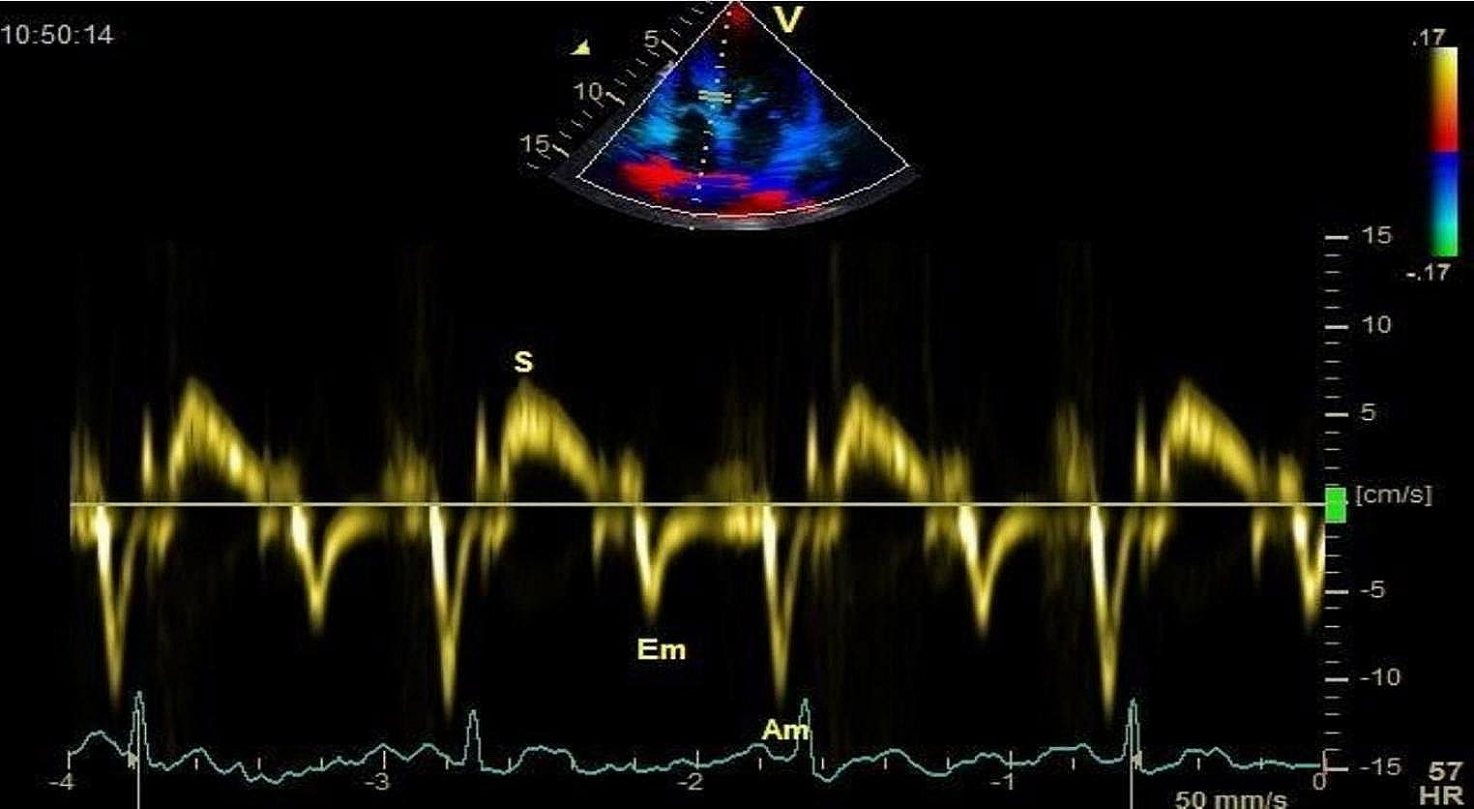
LV diastolic function by TDI

Longitudinal strain and strain rate analysis were performed offline using the ECHOPAC software system. Displacement of each myocardial speckles was tracked and analyzed frame to frame. Three consecutive cardiac cycles were recorded in the apical 4,3,2 chamber views. The LV myocardium was traced in its entirety (Fig. 3).

**Fig. 4:**
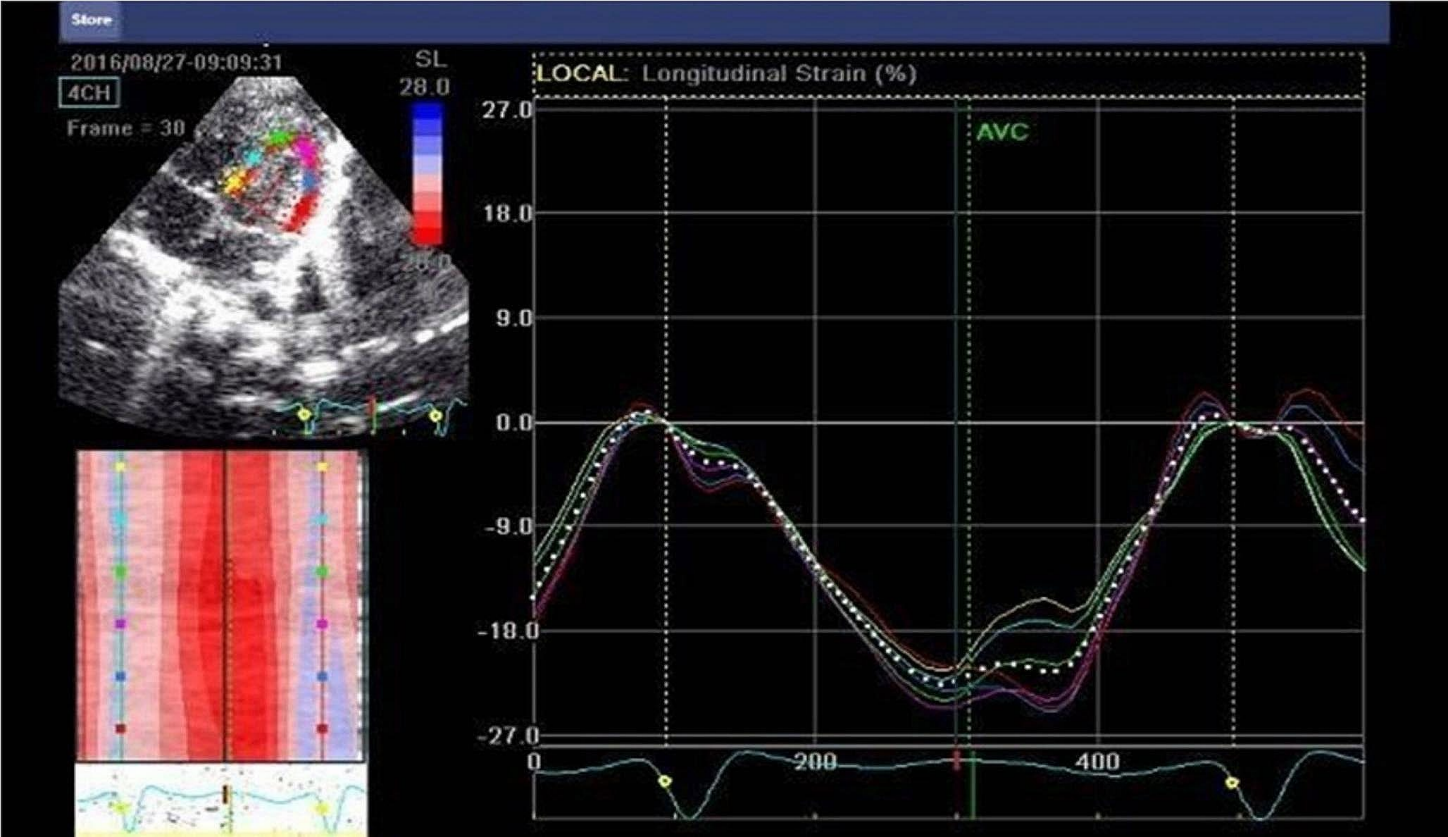
Deformation imaging of LV in apical 4ch view

The software automatically generates a Bull’s eye visualization of 17 myocardial segments, along with strain and strain rate curve patterns for each segment. Strain rates were measured at peak systole, early diastole, and late diastole, and Global Longitudinal Strain (GLS) was obtained [10] (Fig. 4).

### Statistical analysis

All the data was entered in an Excel sheet, and analysis was performed using SPSS version 16. Continuous variables were expressed as mean ± SD. Comparison between cases and controls was done using an independent T test. Non-parametric Kruskal wallis test was used for skewed data and the variables were presented in Median (IQR). Within group comparison was carried out using ANOVA and Post hoc analysis by Bonferroni test. *P* < 0.05 was considered statistically significant.

## Results

### Patient characteristics

A total of 68 neonates were enrolled, of whom 31 were cases and 37 were controls. Among the case subjects, 21 (30.8%) were preterm neonates, while 10 (14.7%) were term neonates. The mean gestational age at birth of the study participants was 35.5 ± 3.6 weeks and the mean age of enrolled neonates was 7.3 ± 5.4 days. Of the 68 neonates, 33 (48.5%) were females and 35 (51.5%) were male.

**Fig. 5:**
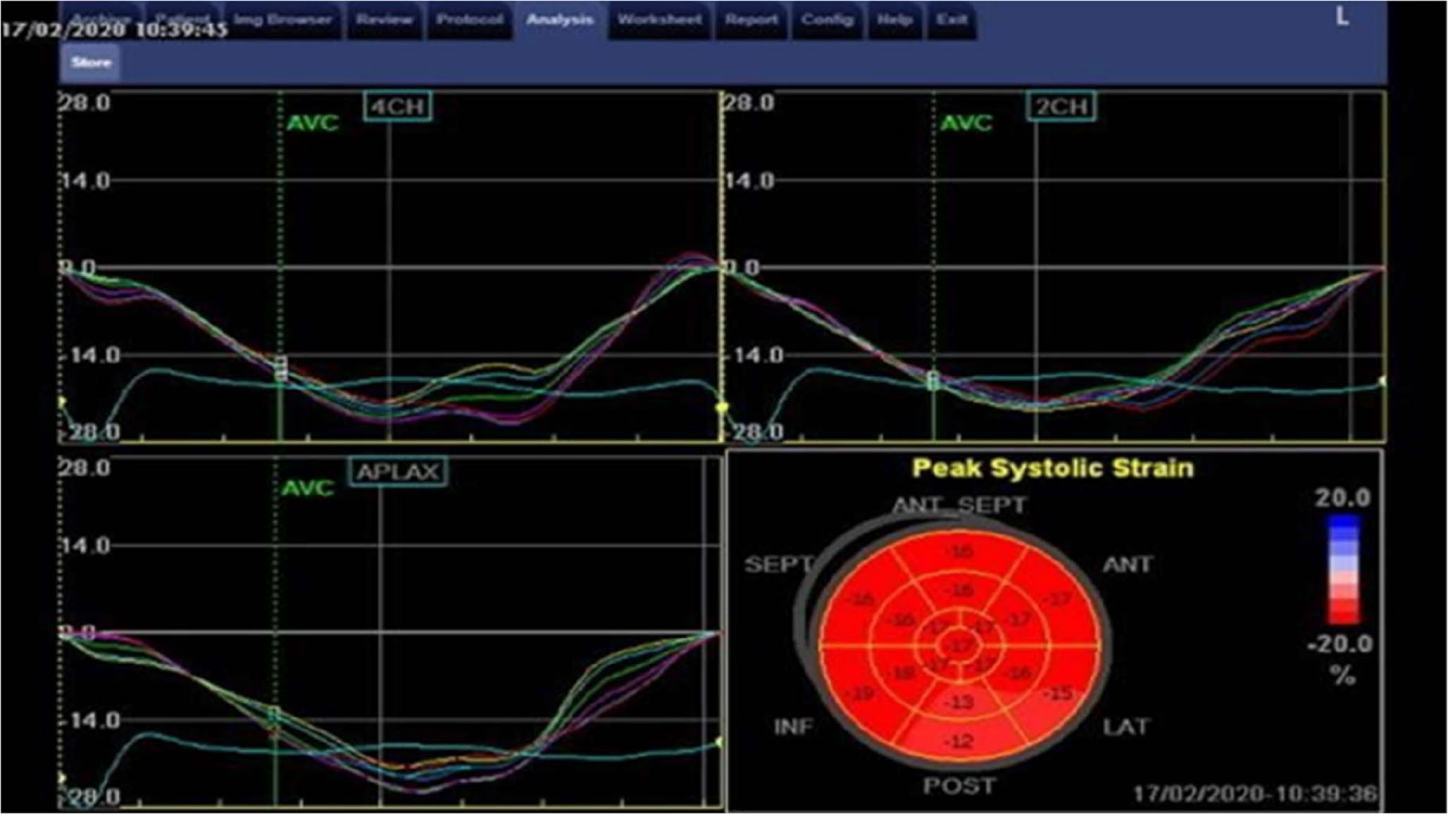
Global longitudinal assessment using STE with bull’s eye model

Group-wise gender analysis revealed that among the preterm neonates, 14 (66.7%) were male and 7 (33.3%) were female. Among the term neonates, 4 (40%) were male and 6 (60%) were female. Among the control group, 17 (45.9%) were male and 20 (54.1%) were female (Fig. 5).

Other demographic variables and clinical characteristics are mentioned in (Table 1).

**Table 1:** Demographic variables and clinical characteristics of the subjects

Variable	Preterm *N* = 21 [Mean ± SD] OR [Median (IQR)]	Term *N* = 10 [Mean ± SD] OR [Median (IQR)]	Control *N* = 37 [Mean ± SD] OR [Median (IQR)]	*P* Value
Age in days	9 (6.5, 14.5)	9 (5,19)	4(2, 6)	< 0.001*
Length (cm)	40 ± 3.6	47.7 ± 2.9	46.3 ± 3.6	< 0.001*
Birth weight (g)	1481 ± 433	2674 ± 305.7	2674 ± 486.5	< 0.001*
Gestational age (weeks)	31.6 ± 2.9	39 ± 0.78	37.0 ± 2.0	< 0.001*
Invasive ventilation n (%)	6(28%)	3 (30%)	-	-
Non-invasive ventilation n (%)	13(61%)	3 (30%)	-	-
No respiratory support n (%)	2(9%)	4 (40%)	-	-

Among the 31-culture positive septic neonates in our study, Klebsiella pseudomonas was the predominant pathogen 12 (38.7%) followed by Acinetobacter baumanii (25.8%) and Escherichia coli (16.1%) (Table 2).

**Table 2:** Pathogens involved in sepsis among the study population

Organism	*N* = 31
Klebsiella pseudomonas	12(38.7%)
Acinetobacter baumanii	8 (25.8%)
Escherichia coli	5 (16.1%)
Enterococcus faecium	2 (6.45%)
Streptococcus agalactiae	1 (3.22%)
Herbaspirullum	1 (3.22%)
Streptococcus gallolyticus	1 (3.22%)
Chryobacterium sepsis	1 (3.22%)

Preterm neonates needed more ventilator and non-invasive ventilation (NIV) support than term neonates during hospital stay, with dopamine being the most frequently used inotropic agent.

It was observed that C-reactive protein(CRP) is substantially high in preterm neonates than in term and control neonates with a p value < 0.05 (Table 3).

**Table 3:** Comparison of laboratory parameters among controls and sub group of cases

Lab parameters	Preterm *N* = 21 [Mean ± SD] OR [Median (IQR)]	Term *N* = 10 [Mean ± SD] OR [Median (IQR)]	Control *N* = 37 [Mean ± SD] OR [Median (IQR)]	*P* Value
Hb (g/dl)	15.2 ± 3.6*	15.8 ± 3.1	17.7 ± 2.9*	0.025*
Hct (%)	45.8 ± 8.8*	47.5 ± 9.8	53.7 ± 9.2*	0.009*
TLC (×10^3^/µL) Median (IQR)	8.6 (4.75, 15.6)	10 (5.702, 13.455)	12.68 (9.57, 16.82)	0.084
Neutrophil (%)	51.6 ± 14.5	55.6 ± 19.7	58.7 ± 19.1	0.364
Lymphocyte (%) Median (IQR)	29 (19.5, 38.55)	24.5 (11.52, 44.075)	21.25(13.22, 38.37)	0.518
Monocyte (%) Median (IQR)	8 (5.5, 12.6)	11.15 (8.75, 12.35)	9.5 (7.05, 12.12)	0.407
Eosinophil (%) Median (IQR)	2 (1.0, 5.5)	3.6 (1, 9)	1.5 (0.90, 3.025)	0.140
PLT (×10^3^/µL) Median (IQR)	189 (90.5, 271)	109 (69, 220)	251 (214, 294.5)	0.003*
CRP (mg/L) Median (IQR)	25.15 (0.745, 77.33)	27.34 (2.31, 49.885)	0.6 (0.6, 2.61)	0.001*

Hb: Hemoglobin, HCT: hematocrit, TLC: Total leukocyte count, PLT: Platelet count CRP: C reactive protein

The echocardiographic parameters of septic new-borns and control group are summarized in Tables 4 and 5.

**Table 4:** Comparison of echocardiographic parameters between cases and controls

Parameters	Case *N* = 31	Control *N* = 37	*P* value
IVSd (mm)	3.35 ± 0.79	3.54 ± 0.60	0.279
IVSs (mm)	3.54 ± 0.92	4.05 ± 0.8	0.022*
LVPWd (mm)	3.09 ± 0.78	3.02 ± 0.49	0.660
LVPWs (mm)	4.67 ± 1.04	4.59 ± 0.76	0.707
Mitral E (m/s)	0.50 ± 0.11	0.48 ± 0.12	0.519
Mitral A(m/s)	0.52 ± 0.14	0.42 ± 0.10	0.001*
Mitral E/A ratio	1.01 ± 0.35	1.18 ± 0.31	0.048*
Tricuspid E (m/s)	0.43 ± 0.13	0.43 ± 0.11	0.921
Tricuspid A(m/s)	0.51 ± 0.12	0.470 ± 0.11	0.123
Tricuspid E/A ratio	0.88 ± 0.32	0.95 ± 0.28	0.327
LV EDD (mm)	14.8 ± 2.77	16.54 ± 2.14	0.007*
LV ESD (mm)	9.64 ± 1.74	10.70 ± 1.43	0.008*
LV EF (%)	67.7 ± 5.67	67.83 ± 4.44	0.959
LV FS (%)	34.6 ± 4.16	34.89 ± 3.35	0.788
TAPSE (mm)	9.2 ± 1.84	9.51 ± 1.21	0.553
PASP (mmHg)	21.83 ± 12.06	15.24 ± 3.78	0.002*
LV IVCT (ms)	43.29 ± 6.43	44.18 ± 6.73	0.578
LV IVRT (ms)	46.74 ± 7.65	48.54 ± 8.01	0.350
LV ET (ms)	165.58 ± 24.37	181 ± 23.97	0.011*
LV MPI	0.54 ± 0.09	0.51 ± 0.1	0.221
LV DT (ms)	85.58 ± 29.95	103.62 ± 33.51	0.023*
RV IVCT (ms)	40.51 ± 8.78	42.29 ± 8.53	0.401
RV IVRT (ms)	40.83 ± 8.05	43.02 ± 8.42	0.280
RV ET (ms)	163.8 ± 53.67	187.73 ± 40.71	0.041*
RV MPI	0.54 ± 0.21	0.47 ± 0.14	0.103
RV DT (ms)	90.5 ± 30.68	106.13 ± 38.39	0.073
Septal E’ (m/s)	0.053 ± 0.013	0.051 ± 0.013	0.449
Septal A’ (m/s)	0.054 ± 0.013	0.047 ± 0.012	0.031*
Septal S’ (m/s)	0.04 ± 0.008	0.04 ± 0.007	0.113
Lateral E’ (m/s)	0.062 ± 0.018	0.066 ± 0.016	0.299
Lateral A’ (m/s)	0.067 ± 0.017	0.06 ± 0.013	0.069
Lateral S’ (m/s)	0.055 ± 0.013	0.054 ± 0.008	0.522
RV E’ (m/s)	0.07 ± 0.02	0.07 ± 0.01	0.508
RV A’ (m/s)	0.09 ± 0.017	0.09 ± 0.013	0.668
RV S’ (m/s)	0.07 ± 0.016	0.06 ± 0.01	0.013*
LV STRAIN (%)	-18.8 ± 3.29	-19.58 ± 2.14	0.245
LV SSR (%)	-2.21 ± 0.36	-1.96 ± 0.26	0.002*
LV EDSR	2.87 ± 0.75	3.02 ± 0.61	0.388
LV LDSR	2.59 ± 0.53	1.96 ± 0.47	< 0.001*
RV STRAIN (%)	-23.02 ± 5.38	-21.9 ± 4.26	0.342
RV SSR (%)	-2.57 ± 0.58	-2.09 ± 0.42	< 0.001*
RV EDSR	3.37 ± 1.3	2.63 ± 1.00	0.010
RV LDSR	3.1 ± 1.19	2.55 ± 1.09	0.035

LVEDD-LV internal dimension in diastole, LVESD-LV internal dimension in systole, EF-Ejection fraction FS- Fractional shortening IVSd- septal thickness diastole IVSs- septal thickness systole PWd- posterior wall thickness diastole PWs- posterior wall thickness systole TAPSE-Tricuspid annular plane septal excursion. PASP- pulmonary artery systolic pressure IVCT- Isovolumic contraction time IVRT- Isovolumic relaxation time ET- Ejection time MPI: Myocardial performance index DT- Deceleration time SSR- strain rate EDSR- Early diastolic strain rate LDSR- Late diastolic strain rate

**Table 5:** Comparison of echocardiographic parameters between sub group of cases and controls

Parameters	Preterm *n* = 21	Term *N* = 10	Control *N* = 37	*P* value
IVSd (mm)	3.04 ± 0.58*$	4 ± 0.81*	3.54 ± 0.6$	0.001*
IVSs (mm)	3.14 ± 0.65*$	4.4 ± 0.84*	4.05 ± 0.84$	< 0.001*
LVPWd (mm)	2.81 ± 0.6*	3.7 ± 0.82*$	3.02 ± 0.49$	0.001*
LVPWs (mm)	4.42 ± 0.97	5.2 ± 1.03	4.59 ± 0.76	0.074
Mitral E (m/s)	0.46 ± 0.1*	0.58 ± 0.1*	0.48 ± 0.12	0.042*
Mitral A(m/s)	0.52 ± 0.14*	0.53 ± 0.14$	0.42 ± 0.1*$	0.004*
Mitral E/A ratio	0.94 ± 0.25*	1.18 ± 0.47	1.18 ± 0.31*	0.023*
Tricuspid E(m/s)	0.42 ± 0.11	0.45 ± 0.18	0.43 ± 0.11	0.904
Tricuspid A(m/s)	0.51 ± 0.12	0.53 ± 0.13	0.47 ± 0.11	0.279
Tricuspid E/A ratio	0.87 ± 0.26	0.9 ± 0.42	0.95 ± 0.28	0.602
LV EDD (mm)	13.9 ± 2.66*$	16.9 ± 1.79$	16.54 ± 2.14*	< 0.001*
LV ESD (mm)	9 ± 1.61*$	11 ± 1.15$	10.7 ± 1.43*	< 0.001*
LV EF (%)	67.95 ± 5.8	67.4 ± 5.69	67.83 ± 4.44	0.959
LV FS (%)	34.61 ± 4.27	34.7 ± 4.16	34.89 ± 3.35	0.963
TAPSE (mm)	8.61 ± 1.28*	10.7 ± 2.11*	9.51 ± 1.21	0.001*
PASP (mmHg)	21 ± 12.33	23.6 ± 11.89*	15.24 ± 3.78*	0.008*
LV IVCT (ms)	42.85 ± 6.58	44.2 ± 6.33	44.18 ± 6.73	0.747
LV IVRT (ms)	46.52 ± 7.92	47.2 ± 7.42	48.54 ± 8.01	0.633
LV ET (ms)	163.61 ± 27.21*	169.7 ± 17.51	181 ± 23.97*	0.032*
LV MPI	0.55 ± 0.1	0.53 ± 0.08	0.51 ± 0.1	0.446
LV DT (ms)	79.52 ± 26.78*	98.3 ± 33.64	103.62 ± 33.51*	0.024*
RV IVCT (ms)	40.81 ± 9.45	39.9 ± 7.6	42.29 ± 8.53	0.679
RV IVRT (ms)	40.76 ± 8.39	41 ± 7.71	43.02 ± 8.42	0.559
RV ET (ms)	162.66 ± 54.97	166.2 ± 53.62	187.73 ± 40.71	0.123
RV MPI	0.54 ± 0.20	0.54 ± 0.25	0.47 ± 0.14	0.268
RV DT (ms)	84.09 ± 30.11	104.1 ± 28.66	106.13 ± 38.39	0.067
Septal E’ (m/s)	0.05 ± 0.012	0.06 ± 0.014	0.051 ± 0.013	0.130
Septal A’ (m/s)	0.051 ± 0.014	0.060 ± 0.012*	0.047 ± 0.012*	0.026*
Septal S’ (m/s)	0.044 ± 0.008	0.05 ± 0.009	0.043 ± 0.007	0.052
Lateral E’ (m/s)	0.054 ± 0.013*$	0.078 ± 0.018*	0.066 ± 0.016$	0.001*
Lateral A’ (m/s)	0.065 ± 0.018	0.072 ± 0.013	0.060 ± 0.013	0.110
Lateral S’ (m/s)	0.054 ± 0.015	0.058 ± 0.009	0.054 ± 0.008	0.616
RV E’ (m/s)	0.066 ± 0.021*	0.088 ± 0.025*	0.070 ± 0.015	0.016*
RV A’ (m/s)	0.09 ± 0.013	0.09 ± 0.024	0.09 ± 0.013	0.766
RV S’ (m/s)	0.07 ± 0.013$	0.08 ± 0.018*$	0.06 ± 0.010*	0.001*
LV STRAIN (%)	-18.29 ± 3.64	-19.88 ± 2.17	-19.58 ± 2.14	0.161
LV SSR	-2.19 ± 0.31*	-2.26 ± 0.47$	-1.96 ± 0.26*$	0.007
LV EDSR	2.71 ± 0.8	3.21 ± 0.52	3.02 ± 0.61	0.114
LV LDSR	2.55 ± 0.43*	2.67 ± 0.72$	1.96 ± 0.47*$	< 0.001
RV STRAIN (%)	-22.16 ± 5.6	-24.82 ± 4.65	-21.9 ± 4.26	0.226
RV SSR	-2.59 ± 0.65*	-2.52 ± 0.44	-2.09 ± 0.42*	0.001
RV EDSR	3.3 ± 1.08	3.52 ± 1.73	2.63 ± 1.00	0.32
RV LDSR	3.04 ± 1.11	3.38 ± 1.4	2.55 ± 1.09	0.082

LVEDD-LV internal dimension in diastole, LVESD-LV internal dimension in systole, EF-Ejection fraction FS- Fractional shortening IVSd- septal thickness diastole IVSs- septal thickness systole PWd- posterior wall thickness diastole PWs- posterior wall thickness systole TAPSE-Tricuspid annular plane septal excursion. PASP- pulmonary artery systolic pressure IVCT- Isovolumic contraction time IVRT- Isovolumic relaxation time ET- Ejection time MPI: Myocardial performance index DT- Deceleration time SSR- strain rate EDSR- Early diastolic strain rate LDSR- Late diastolic strain rate

* and $ indicate that the observed differences between groups are statistically significant (*p* < 0.05)

The Ejection Fraction and fractional Shortening determining LV systolic function showed no significant difference between septic preterm, term and control groups. LV internal dimensions are higher in term neonates than preterm neonates which was statistically significant (*p* < 0.05). Mitral E/A ratio is lower in septic newborns than controls. Inexplicably, non-septic control newborns had lower mitral E (0.48 ± 0.12 vs. 0.58 ± 0.1) and A wave (0.42 ± 0.1 vs. 0.53 ± 0.14, *p* < 0.05) velocities when compared with septic term neonates.

TAPSE is significantly lower in preterm neonates indicating impaired RV systolic function while substantially high Pulmonary artery systolic pressure (PASP) was noted in two preterm and one term septic newborns. A positive correlation was noted between CRP and PASP, however, other echocardiographic parameters did not show any correlation. LV and RV ejection time, LV deceleration time were significantly lower in neonates with sepsis than in non-septic neonates with a p value < 0.05. However, the Myocardial Performance Index (MPI) showed no difference between septic neonates and control group.

The septal A’ velocity and RV S’ velocity was higher in neonates with sepsis than in controls, which was statistically significant (p value < 0.05). Other parameters, however, displayed no significant variation between groups.

In comparison to controls, the LV myocardial contractility is slightly reduced in septic neonates. However, LV systolic strain rate (SSR), LV late diastolic strain rate (LDSR), RV Systolic Strain Rate were significantly higher in septic newborns than in controls (*p* < 0.05).

In our investigation comprising 31 neonates with sepsis, we observed a mortality rate of 12.9%, with 4 neonates succumbing to death. Septic shock emerged as the predominant cause of mortality among the affected neonates.

## Discussion

Increased neonatal vulnerability to sepsis is primarily due to an immature immune system. During the period of sepsis in neonates, the cytokine production, as well as the concomitant acidosis and hypoxia, may be responsible for the myocardial dysfunction [11–13].

In the present study a total of 68 neonates which included [33 female and 35 males], with a mean age of 7.3 ± 5.4 days were enrolled.

In the present study, CRP is markedly higher in neonates with sepsis than in the control group. Moreover, preterm neonates exhibited higher levels of CRP than term babies. According to the literature, CRP can be deemed a dependable adjunctive tool for suspecting sepsis, despite its nonspecific nature [14] [Table 3].

As a measure of systolic function, LV fractional shortening is commonly applied to neonates. However, it is highly sensitive to variations in heart rate and ventricular burden [15]. As a measure of global left ventricular systolic function, Systolic velocity wave (Sm) of mitral annulus is preferred as it is less sensitive to loading conditions [16]. The results of our study revealed no statistically significant distinction in LV systolic function and Sm of the mitral annulus when comparing the septic group to the control group, as well as between preterm and term neonates. These findings align with a previous study conducted by Tomerak RH et al. [17] and differ from those of the earlier study conducted by Abdel- Hady et al. who demonstrated that septic neonates showed significantly lower Sm values compared to controls suggesting LV systolic dysfunction [18]. 

Diastolic dysfunction is characterized by impaired ventricular filling and relaxation during diastole. The findings of our study revealed that the E/A ratio of the mitral valve was significantly lower in septic newborns than in healthy neonates, suggesting left ventricular diastolic dysfunction which is in accordance with the findings of Tomerak et al. [17]

On the contrary, Abdel-Hady et al. found no statistically significant difference in mitral E/A ratio among septic and healthy neonates [8]. 

On comparison of diastolic parameters among preterm and term neonates with sepsis, mitral E wave velocity and E/A ratio are lower in preterm neonates. It’s because of poor cardiac compliance and alleviated diastolic performance.

Preterm newborns have lower trans-mitral early filling flow phase (E- wave) than active flow (A-wave). As a result, the E/A ratio is less than 1.0. This is in contrast to the term neonate, in which the passive flow phase predominates and the E/A > 1 [18]. These findings are in contrast with those of Tomerak et al who reported no significant difference in mitral E and E/A ratio between term and preterm septic neonates. The results of our study suggest that preterm neonates showed significantly lower lateral E’ and RV E’ velocities than term neonates may be indicating an early impaired LV and RV diastolic dysfunction respectively.

The current study revealed that pulmonary artery systolic pressure was substantially higher in septic newborns than in controls (Table 5). This is in support of the previous findings by Mohsen and Amin, who disclosed that neonatal sepsis is the second most prevalent cause of pulmonary hypertension, accounting for 43.7% of all cases [19].

We found no significant variations in the E/A ratio of the tricuspid valve among septic and healthy newborns, which is in accordance with the findings of [8].

A study conducted by Abdel-Hadey et al., 2012 aimed at analysing myocardial performance using tissue doppler imaging reported a significant increase in Myocardial Performance Index in neonates with sepsis. However, in the present study the Myocardial Performance Index showed no significant difference between the septic neonates and controls, which was partially consistent with the findings of Tomerak et al. The differences in findings may be due to unequal sample distribution. Abdel hadey et al., also stated that septic newborns had significantly lower RV S’ velocity when compared to healthy controls, indicating right ventricular systolic dysfunction [8].

TAPSE is a simple method that is confirmed to be relevant for assessing right ventricular function, and in our study, when TAPSE was compared among term and preterm septic neonates, it was significantly lower in septic preterm neonates, suggesting impaired RV systolic function (Table 5).

Speckle tracking echocardiography is superior to Tissue Doppler Imaging in providing an angle-independent evaluation of regional myocardial deformation [20]. In our study, the LV global longitudinal strain was slightly lower in preterm septic neonates than in term neonates with sepsis, may be indicating subclinical myocardial dysfunction which is in line with the study conducted by Hirose A et al [21].

On the contrary, Mostafa Awany et al., reported significant reductions in LV and RV Global Longitudinal Strain values among septic neonates than healthy neonates indicating sub clinical systolic dysfunction [22].

According to a study conducted by Hirose et al., preterm newborns had lower baseline circumferential early diastolic strain rate and greater late diastolic strain rate than healthy neonates. And also, there were no statistically significant differences between preterm infants and controls in terms of Ejection fraction, Fractional Shortening, longitudinal or circumferential strain, or strain rate [21].

In our study we found that LV Systolic strain rate and RV Systolic strain rate showed statistically significant difference between cases and controls with higher values in septic neonates however strain rate values cannot clinically detect cardiac dysfunction in neonates with sepsis [Table 4].

Further studies are needed to determine the optimal approach to strain imaging in neonates with sepsis, and to validate its use in this population.

### Limitations

Unequal sample distribution among preterm and term neonates with sepsis is the major limitation of our study. Due to technical issues, time frame and challenging echo windows in extremely preterm, very low birth weight neonates the sample size could not be achieved. And parents of premature or critically sick infants did not give their consent for this study.

## Conclusion

Upon the assessment of cardiac function by conventional, Tissue Doppler Imaging and speckle tracking echocardiography, it is found that septic newborns are associated with LV diastolic dysfunction, RV systolic dysfunction, and substantially higher pulmonary systolic pressures.

## Data Availability

Master sheet with individual patient data will be submitted if requested.
